# Thermo-mechanical behavior measurement of polymer-bonded sugar under shock compression using in-situ time-resolved Raman spectroscopy

**DOI:** 10.1038/s41598-022-05834-3

**Published:** 2022-02-03

**Authors:** Abhijeet Dhiman, Nolan S. Lewis, Ayotomi Olokun, Dana D. Dlott, Vikas Tomar

**Affiliations:** 1 School of Aeronautics and Astronautics, Purdue University, ARMS 2033, 701 W. Stadium Ave., West Lafayette, IN 47907-2045 USA; 2 School of Chemical Sciences and Fredrick Seitz Materials Research Laboratory, University of Illinois at Urbana-Champaign, Urbana, IL 61801 USA

**Keywords:** Characterization and analytical techniques, Mechanical engineering

## Abstract

Quantitative information regarding the local behavior of interfaces in an inhomogeneous material during shock loading is limited due to challenges associated with time and spatial resolution. This paper reports the development of a novel method for in-situ measurement of the thermo-mechanical response of polymer bonded sugar composite where measurements are performed during propagagtion of shock wave in sucrose crystal through polydimethylsiloxane binder. The time-resolved measurements were performed with 5 ns resolution providing an estimation on local pressure, temperature, strain rate, and local shock viscosity. The experiments were performed at two different impact velocities to induce shock pressure of 4.26 GPa and 2.22 GPa and strain rate greater than 10^6^/s. The results show the solid to the liquid phase transition of sucrose under shock compression. The results are discussed with the help of fractography analyses of sucrose crystal in order to obtain insights into the underlying heat generation mechanism.

## Introduction

Polymer bonded explosives (PBXs) or their mock composites have a heterogeneous microstructure that contains a high concentration of crystals in a polymeric binder. In order to reduce the sensitivity of such materials to mechanical and thermal stimuli, the crystals are embedded inside soft polymers such as hydroxyl-terminated polybutadiene (HTPB) and polydimethylsiloxane (PDMS). The mismatch in properties between phases leads to a complex shock-induced hot spot initiation mechanism at the mesoscale where particle chemistry, inter-particle interactions, and mechanics of different phases and interfaces play a crucial role. The mechanism of shock-induced hot spot initiation, ignition, and detonation of energetic materials is a multiscale phenomenon where a complete understanding of the underlying mechanism requires theoretical and experimental studies over time scales of pico- to nanoseconds and length scales of nano- to micrometers in order to quantify microstructural effects and macroscale reaction propagation^1–9^. The interaction of shock wave with the microstructure of composite material creates pressure and temperature localization with complex spatial profiles at micro and mesoscale. The plastic deformation and fracture of the binder, crystal and interfaces along with void collapse and frictional heating of the failure sites lead to dissipation of the energy in the localized regions. In the case of energetic materials, the localized temperature rise can lead to the formation of hot spots and can subsequently start chemical reactions. Under favorable conditions, such hot spots can lead to critical size and temperature in order to initiate detonation. The hot spot initiation mechanism can be a combination of frictional heating^10,11^, temperature rise in shear bands^12,13^, the interaction of crack surfaces^14–16^, plastic dissipation, and gas compression during void collapse^4,17–21^. The complex interaction between the underlying mechanisms during shock compression makes it difficult to analyze the individual contributions to overall behavior and initiation of energetic materials.

**Figure 1:**
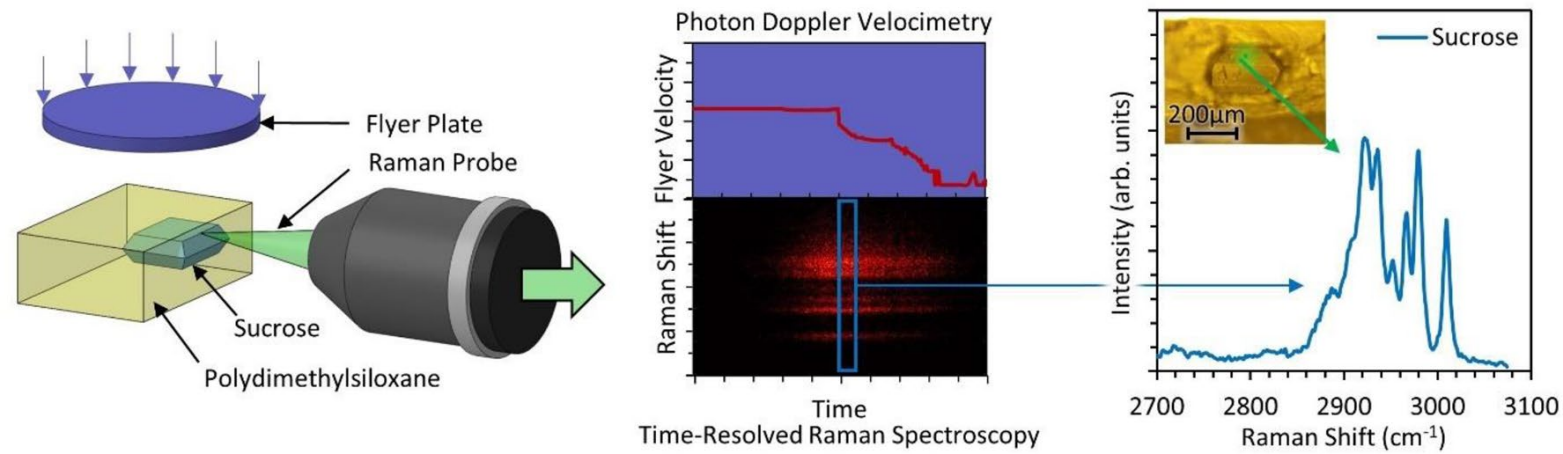
Schematic illustration of experimental concept (not to scale) for in-situ analysis using Raman spectroscopy and photon Doppler velocimetry during shock compression.

Computational and numerical methods have been widely used to study the contribution of individual and combined mechanisms responsible for heat generation in heterogeneous materials. In the case of energetic materials, computational methods have provided an improved understanding of temperature rise due to void collapse^12,18,20^, shear deformation^22,23^, interface failure^24–27^, and friction^11,14,24^. However, the computational methods still require calibration and validation using experiments. Conversely, experiments under shock conditions have provided insights into temperature rise due to gas compression^4,19^ and void collapse^17,21^. Interparticle friction^15,16,24,28,29^ and viscous heating^26,30,31^ effects have been addressed under weak shock conditions. However, the contribution from friction and plastic deformation in microscale domains at shock conditions has not been explored due to the lack of an ability to simultaneously measure stress and temperature under shock conditions. The experimental measurement of the thermo-mechanical response of heterogeneous material under shock conditions at the microstructure level is significantly challenging due to the required spatial and temporal resolution. In this paper, these challenges are addressed through the development of a novel capability for in-situ measurement of both the temperature and pressure over the microscale domain of a composite material. Figure 1 shows a simplified representation of the experimental concept where a flyer plate is impacted on a sucrose sample embedded inside polydimethylsiloxane binder. The measurement of shock response is done using in-situ Raman spectroscopy and photon Doppler velocimetry during the shock compression of microstructure. This work allows performing time-resolved Raman spectroscopy with 5 ns resolution during several hundreds of shock experiments at different impact velocities (0.6–1.2 km/s).

The use of Raman spectroscopy for characterizing shock behavior has been explored by many researchers in the past^3,8,9,32^. In our previous work^2^, shock stress at the HMX-HTPB interface using time-gated Raman spectroscopy was measured. Also, a change in Raman spectra was observed for sucrose under shock compression^33^. Hare et al.^3^ were able to obtain temperature and pressure during pico-second ablation of poly-(methyl methacrylate) thin film by taking effects of temperature and pressure. The Raman shift due to the combined effects of temperature and pressure was calibrated using static high pressure and temperature measurements. However, the temperature measurements were not isochoric resulting in a small error due to thermal expansion. Trott et al.^32^ studied shock compression of triaminotrinitrobenzene and found a small difference in Raman shift under pressure compared to quasi-static experiments. This difference was attributed to temperature rise during shock compression. Similar observations were made by Hebert et al.^8^ with shock experiments on TATB. In this work, we used a similar method as Hare et al.^3^ to separate the effects of temperature and pressure on Raman shift. The effects of temperature and pressure on Raman shift were separated by measuring the change in Raman shift of different functional groups of sucrose which is a widely used energetic material substitute^28,29,34,35^. Using this principle, local shock stress and temperature-induced in the polymer-bonded sugar sample during shock loading are measured.

Along with the measurement of pressure and temperature, time-resolved spectroscopy is also used to measure the local shock pressure rise time^36^. The measured shock rise time is used to estimate viscous stress during the compression of the microscale domain through the characterization of local effective shock viscosity. During shock compression, the strain rate at the shock front is finite and is dependent on shock viscosity. Although, a complete understanding of the reason behind shock viscosity is not available. For a crystalline such as HMX, shear banding due to large velocity gradients is observed as a possible reason for shock viscosity^22,23^. The fracture mechanics of the interface also affects the overall shock viscosity during shock wave rise^7,26,37^. Shear-thinning has been observed in many metals and granular materials with increasing strain rates^38^. Such computational studies also suggest a strong dependency of temperature rise and reaction rate in the shear bands to shock-induced viscous dissipation and shock viscosity. Pressure-dependence of viscosity has also been accounted in few simulation models^22,23^. Therefore, simultaneous measurement of stress, temperature, and strain rate provides a complete description of shock viscosity which can be used to calibrate computational models.

## Results

### Flyer impact characterization

**Figure 2:**
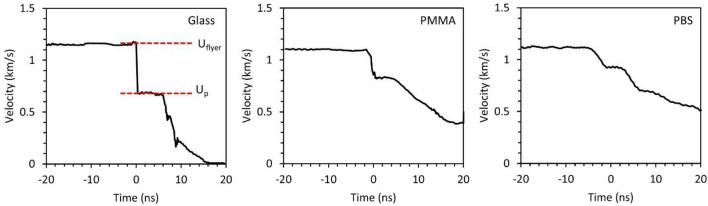
Illustrations of the velocity profiles of 25 μm flyers during travel and the impact on the glass, PMMA, and PBS samples obtained using photon Doppler velocimetry at an impact velocity of 1.2 km/s.

The velocity profile for 25 μm flyers during the impact on the glass, polymethyl methacrylate (PMMA), and polymer-bonded sugar (PBS) samples are shown in Fig. 2. The velocity profile on glass represents the flyer velocity in free space (U_flyer_) and particle velocity at the flyer/glass interface (U_p_) during the impact. A fully supported shock of thickness ~ 5 ns is observed on targets as is typical for a 25 μm aluminum flyer^5^. The shock wave in PMMA and PBS has a similar profile with a higher rise time to reach the shocked state. This behavior can be attributed to the viscoelastic behavior of material compared to a brittle material such as glass where a steeper drop in flyer velocity is observed during shock loading of the target.

Due to the nature of Raman spectroscopy measurements, several hundreds of experiments are required to collect Raman signal with ns resolution. Therefore, variance estimation in the flight time and shock arrival time at the sucrose crystal is important to obtain correct synchronization for the spectroscopy system. From over 100 impacts using 25 μm and 50 μm flyers, the impact velocity and errors (one standard deviation) of 0.62 ± 0.06 km/s and 1.21 ± 0.12 respectively were obtained. The error in impact time on the sample was estimated to be ± 71.6 ns and ± 58.4 ns respectively. The additional error of ~ 3.5 ns in shock arrival time can be estimated due to sample preparation using the speed of sound in PDMS^39^. Therefore, correction between the time of impact on the sample and probing time of Raman spectroscopy is required to obtain a desired resolution of 5 ns. This is achieved using PDV with at least 1 ns resolution in data processing using STFT. The detailed uncertainty analysis of the experiment is given in the supplementary datasheet.

### Effect of laser heating on sucrose crystal

**Figure 3:**
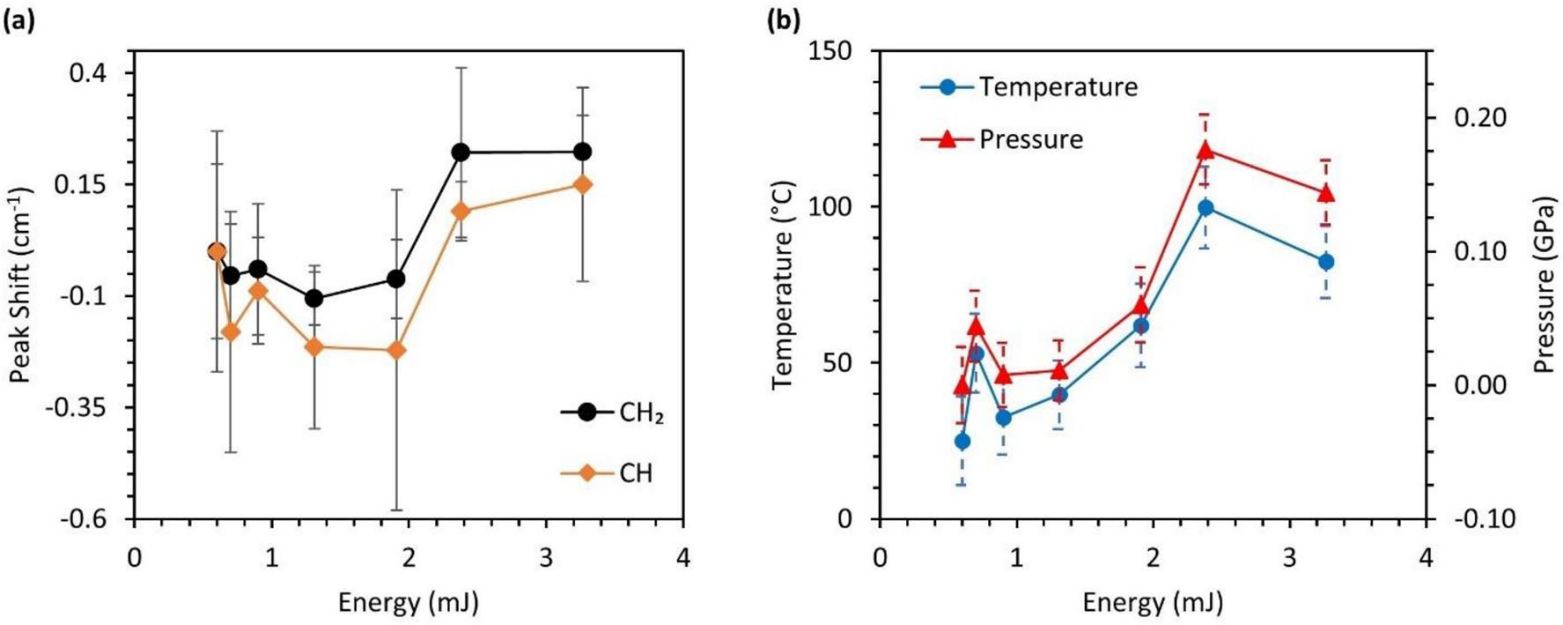
Analysis of the mechanical and thermal effects on sucrose with an increase in probe pulse energy where (**a**) shows the change in Raman shift of CH_2_ and CH functional group in sucrose with laser pulse energy and (**b**) shows the predicted pressure and temperature rise in sucrose crystal with laser pulse energy (20 ns at 10 Hz).

The effect of pulse energy from 532 nm laser on the initial thermodynamic state of sucrose crystal is analyzed using the same approach as described in the later section. To maximize the excitation of the Raman signal, the pulse energy is selected just below the damage threshold. Figure 3a shows the change in Raman shift of the CH_2_ and CH group with increasing laser pulse energy. This data is acquired at 10 Hz. With an initial increase in laser energy, the blue shift of both the functional groups can be observed. With pulse energy higher than 1.9 mJ, the behavior of peaks changes to redshift. This can be explained through temperature and pressure estimated at corresponding laser energies shown in Fig. 3b. Initially, the effects of pressure rise is small compared to temperature, and later with the increase in pressure due to thermal expansion, an increase in redshift can be observed. Beyond the pulse energy of 2.4 mJ, cracks were observed on the sucrose crystal due to rapid thermal expansion from the laser pulse. Therefore, shock experiments were performed at 2.4 mJ to avoid laser damage to the crystal.

### Shock characterization using time-resolved Raman spectroscopy

**Figure 4:**
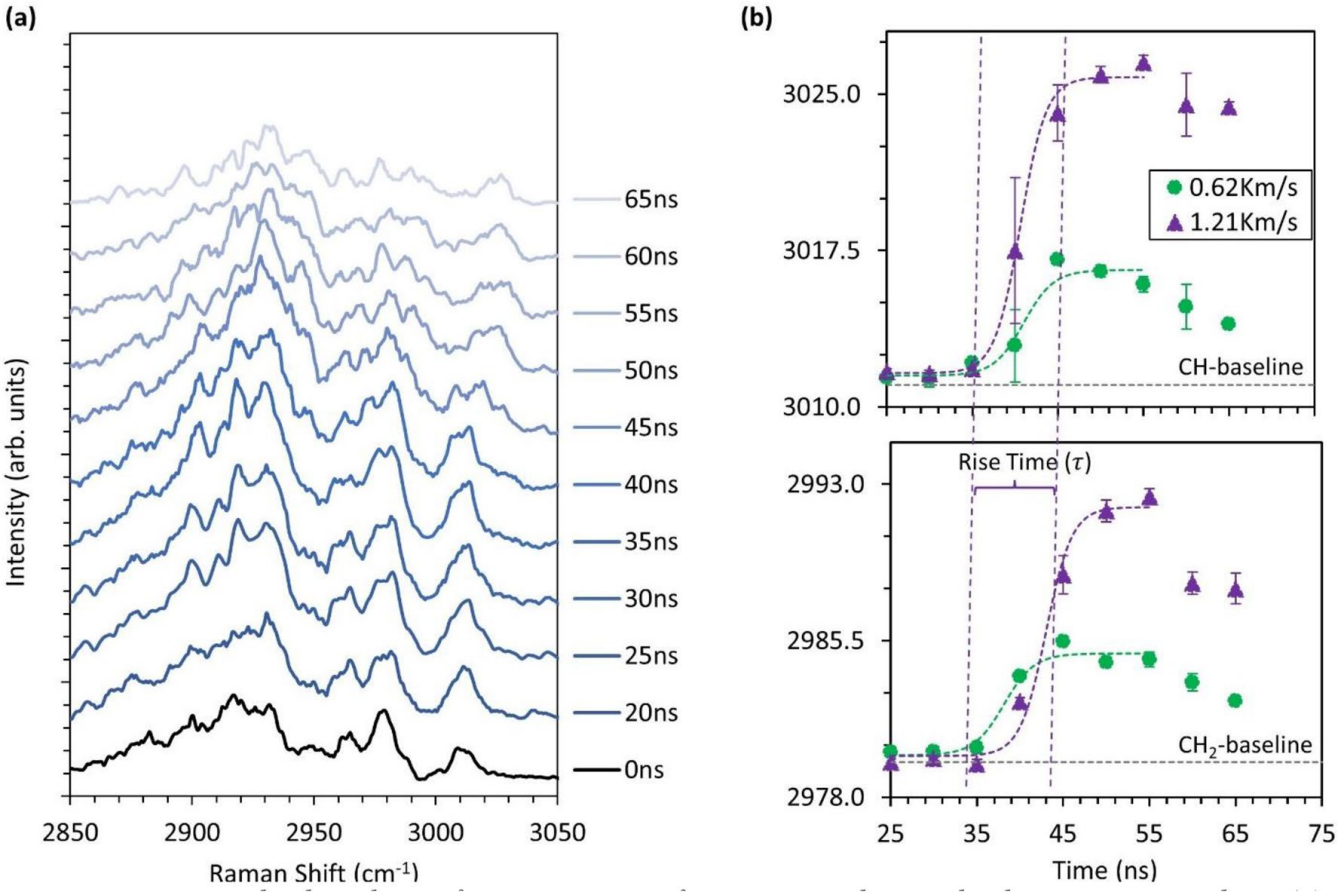
Time-resolved analysis of Raman spectra from sucrose during shock compression where (**a**) shows the time-evolution of Raman spectra of sucrose during impact at 1.21 km/s and (**b**) shows the change in peak location of CH_2_ and CH functional groups in sucrose under shock compression during impact at speeds of 0.62 km/s and 1.21 km/s.

Figure 4a shows the Raman spectra of sucrose during the shock pressure rise from impacts at 1.21 km/s. These spectra were collected from approximately 400 impact experiments.

**Figure 5:**
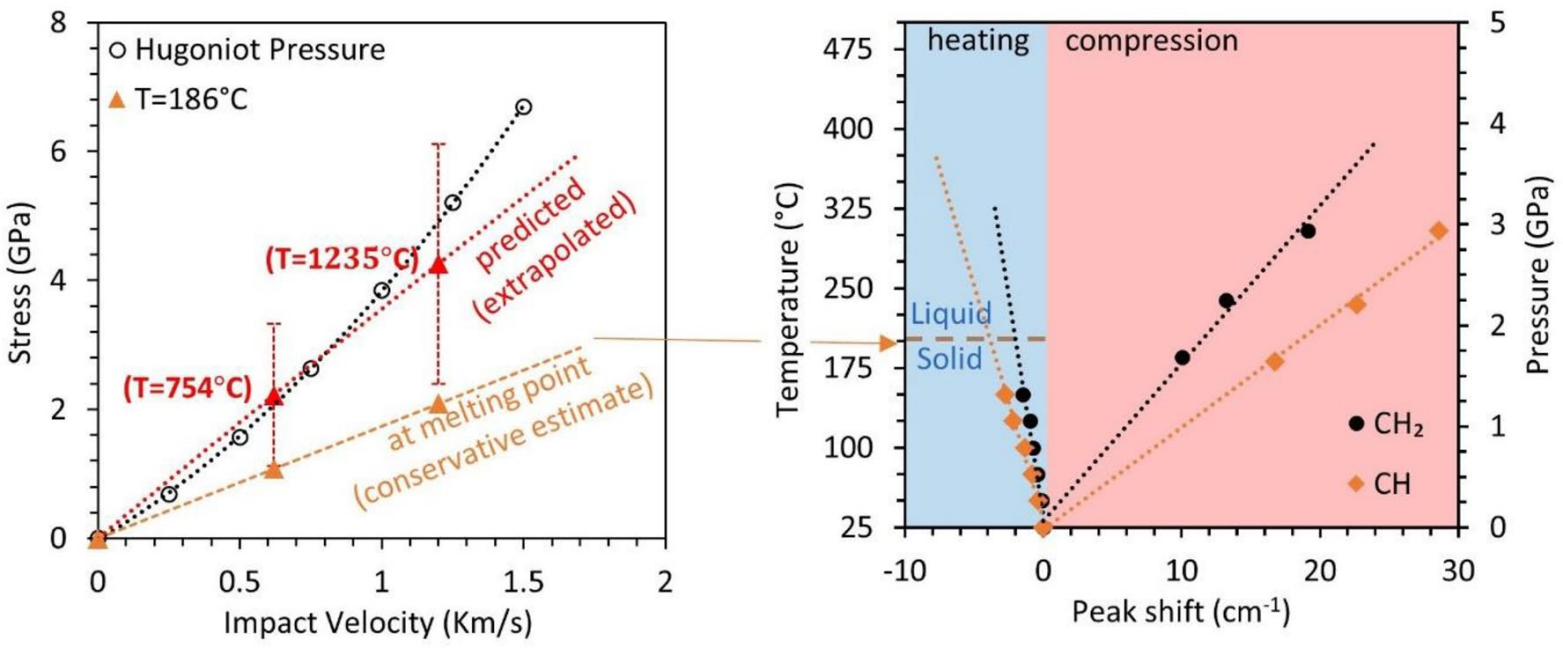
Estimated shock pressures during shock compression at different impact velocities by accounting for temperature effects with different limits on maximum temperature.

The streak data was binned over a 5 ns window to resolve Raman shifts and obtain peak location using Lorentz peak fitting in Origin Pro (OriginLab Corporation). Peak fitting analysis is given in the supplementary datasheet. Several changes in Raman spectra can be observed during the rise of shock pressure. Most noticeable are the broadening and splitting of CH_2_ and CH functional groups to a higher shift at 45 ns after impact. The broadening of Raman shift is associated with the temperature rise during shock compression and the splitting of the peak to a higher shift is associated with the increased pressure. The higher error at 40 ns corresponds to uncertainty in curve fitting during the shock loading of sucrose crystal as shown in Supplementary Fig. S12. The functional groups between 2900 and 2950 cm^−1^ appear to diminish and the combined peaks are observed at 2935 cm^−1^ and 2954 cm^−1^ at around 45–65 ns. Similar behavior is observed at 150 °C for sucrose crystal at ambient pressure where these functional groups form a combined peak at 2915 cm^−1^ and 2941 cm^−1^. The Raman spectra of sucrose as a function of temperature is provided in Supplementary Fig. S9*.* This change is attributed to the phase change of sucrose from solid to liquid, close to the melting point of 186 °C^40^. This behavior was observed for both 0.62 km/s and 1.21 km/s impacts, suggesting a temperature rise higher than the melting point of sucrose. Figure 4b shows the time-resolved Raman shift of CH_2_ and CH functional groups binned over the 5 ns window. The error indicated on the graph is the fitting error of the Lorentz curve. Both the functional groups show redshift under shock compression with higher change for 1.21 km/s impact. A higher change is expected due to higher shock pressure from impact. An estimate on pressure and temperature is performed using Eqs. (3) and (4), the pressure of 2.22 ± 1.1 GPa and 4.26 ± 1.86 GPa, the temperature of 754 °C and 1235 °C is estimated from impact velocity of 0.62 km/s and 1.21 km/s respectively. This further provides evidence of melting observed initially through Raman spectra. However, the estimated temperature is an extrapolation using calibration beyond melting temperature. This estimation of temperature and pressure cannot be verified due to a lack of calibration data beyond the melting point. Due to this limitation, the temperature calculation shows ~ 45% uncertainty and further leads to higher uncertainty in the prediction of pressure. A conservative estimate of pressure is obtained by limiting temperature to the melting point (T = 186 °C) where the pressure of 1.08 ± 0.05 GPa and 2.09 ± 0.7 GPa is estimated. The Hugoniot shock pressure in Fig. 5 is estimated using impedance matching between aluminum 1100 flyer^39^, PDMS binder^39^, and sucrose crystal^34,35^.

The estimation of the strain rate and shock viscosity is done as follows. The rise time of steady shock wave is related to strain rate and Hugoniot state stress^38^ as

1 $$\tau \approx \frac{{\sigma_{h} }}{{\rho C_{0}^{2} \dot{\varepsilon }}}$$

The rise time ($\tau$) of the shock front was obtained through Boltzmann fit between the initial state of Raman shift and final state at maximum stress as shown in Fig. 4*.* The first derivative of Boltzmann fit gives a Gaussian distribution and the rise time is taken as three times the standard deviation of Gaussian distribution. This estimate provides about 99.7% of the total curvature of the shock front. Using sound speed ($C_{o}$) as 3.04 km/s and density ($\rho$) as 1.58 g/cm^3^ for sucrose crystal^34,35^, the estimated strain rate and viscosity are given in Table 1.

**Table 1:** Estimated strain rate and shock viscosity.

Impact speed (Km/s)	Pressure (GPa)	Rise time (ns)	Strain rate (10^6^/s)	Shock viscosity (Pa s)
0.62	1.08 ± 0.05	8.16	9.06	4.5
1.21	2.09 ± 0.08	6.71	21.3	7.2

**Figure 6:**
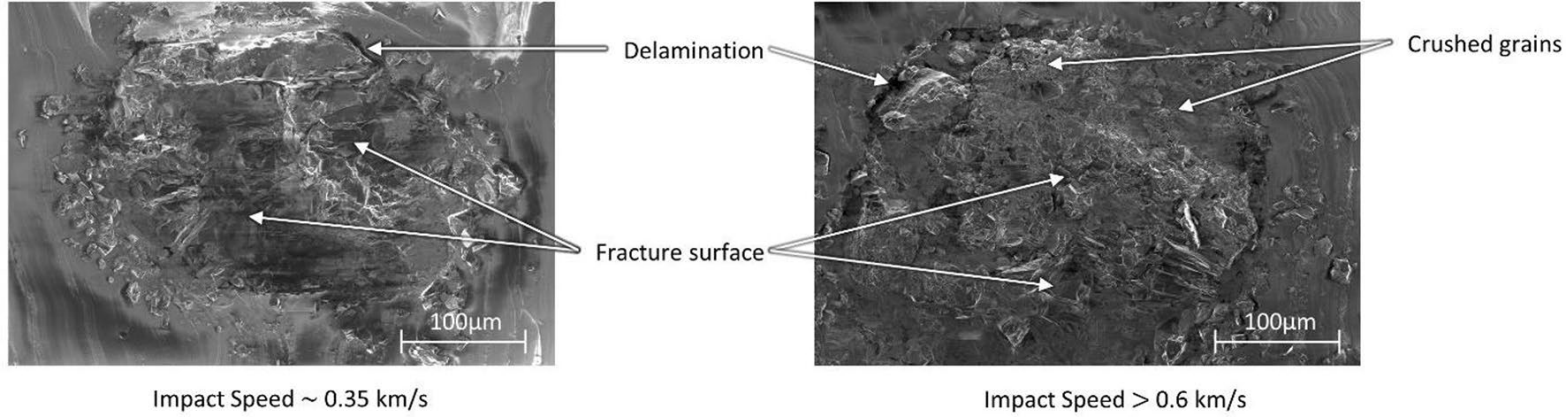
Scanning electron microscopy images of sucrose crystal post-impact at 0.35 km/s and higher than 0.6 km/s. The increase in impact velocity shows severe crushing and compaction of sucrose crystal.

Under shock compression, the sucrose crystal undergoes fracture at different scales. Figure 6 shows SEM images of sucrose crystal to get insight into the heat generation mechanism. On the left is the crystal with impact at speeds ~ 0.35 km/s. The image shows fragmentation of crystal into pieces (> 100 µm) but without any severe crushing of the crystal. On the right is the crystal with features observed for 0.62 km/s and 1.21 km/s impact. This figure shows fragmentation into smaller pieces and briquetting of sucrose crystal under shock compression. Fragments of size typically below 10 µm are observed near the upper interface between PDMS and sucrose crystal where in-situ measurements are performed using Raman spectroscopy. The severe crushing of crystal structure and friction between these small crushed grains is believed to be the primary source of temperature rise.

## Discussion and conclusion

In this work, in-situ time-resolved Raman spectroscopy is used to directly measure temperature and pressure during shock compression of polymer bonded sucrose crystal. The use of Raman spectroscopy provides insight into the chemical changes during shock compression and phase change due to temperature and pressure rise. Temperature and pressure are estimated from analysis of change in the shift for two functional groups of sucrose crystals based on calibrated behavior under quasi-static experiments. Time-resolved Raman spectroscopy analysis shows a significant change in vibrational modes indicating features close to the melting point of sucrose. The Raman spectrum under shock compression is comprised of the effects of pressure, plastic deformation and temperature rise. The effect of pressure can be observed from the shift of spectra to higher wavenumber. The extensive deformation of crytal during shock compression leads to inhomogenous material in probed volume. The collection of Raman spectrum over several samples also adds to the inhomogeneity in probed volume. Therefore, subtle differences can be expected between the Raman spectra of molten phase under shock compression and quasi-static heating. This observation is used to correct the estimation of shock pressure. The measured shock pressure and shock rise time are used to characterize shock viscosity which is found to be around ~ 5 Pa·s for strain rate greater than 10^6^/s. A better correction on shock pressure can be done by knowing the behavior of functional groups above melting point, but the weak spectra of CH_2_ (2982 cm^−1^) and CH (3011 cm^−1^) functional groups near melting point limits this analysis. In principle, this method can be extended to energetic materials or other surrogates to study the chemical changes under shock compression.

It is important to note that there can be multiple sources of error between estimated pressure based on impact velocity and pressure estimated from Raman spectroscopy. Firstly, the isobaric temperature and isothermal pressure calibration is not the ideal solution for Raman analysis under shock compression experiments. During the isobaric heating, the material undergoes temperature rise and thermal expansion. As a result Raman shift is affected by both phenomena^41^. The isochoric heating of material is required to obtain ideal calibration of Raman shift with temperature. Such calibration is very difficult to measure instrumentally. Therefore, the estimated pressure and temperature is neglecting the effects of material compressibility on the Raman shifts. The other source of errors includes experiment configuration being not ideally uniaxial planar shock conditions as samples are asymmetric around the axis of impact and the morphology of crystal can lead to shock reflections and multiple interactions. The measured pressure is expected to be lower than Hugoniot state pressure due to the interaction of release waves on this free boundary. Secondly, the experiment configuration can form unsupported shock conditions. Also, temperature-induced phase change due to melting and pressure-induced phase change around 5 GPa in sucrose^42^ leads to error in estimation in pressure and temperature. Finally, the current experiments require correction for the arrival time of shock wave due to inconsistent time of impact at the sample surface. This leads to the addition of Raman spectra over several pulses offset in the time domain. Therefore the temporal effect of laser energy on sucrose crystal cannot be corrected and leads to uncertainties in temperature and pressure calculations. The effect of these limitations will be explored in our future work by performing experiments on a material with a higher melting point and probe laser with long pulse duration.

## Experiment method

### Sample preparation

The microstructure of composite material such as PBX is complex. This limits the repeatability of preparing samples with similar local features and morphologies. To overcome this challenge, a simplified sample structure with a single crystal of sucrose embedded inside the polydimethylsiloxane binder (PDMS) with a controlled orientation was used. Sucrose has been widely used in the energetic materials research community^28,29,34,35,43–45^ as a mechanical simulant of cyclotetramethylene-tetranitramine (HMX) due to its monoclinic crystal structure and similar morphological characteristics. Polymer-bonded sucrose (PBS) which acts as an inert surrogate of polymer-bonded explosive is used to study the effects of morphology and interparticle interactions due to similar shock Hugoniot as HMX^45^ and similar dynamic behavior under impact^44^. The samples were created in this work using single-crystal sucrose particles with an average crystal size of 250 μm. The sucrose crystals were embedded inside the PDMS binder (Sylgard 184, Dow Chemical Company) with an average depth of 106.6 μm and a standard deviation of 10.5 μm. Further details on sample preparation are included in the supplementary datasheet.

### Laser-based projectile launch setup

**Figure 7:**
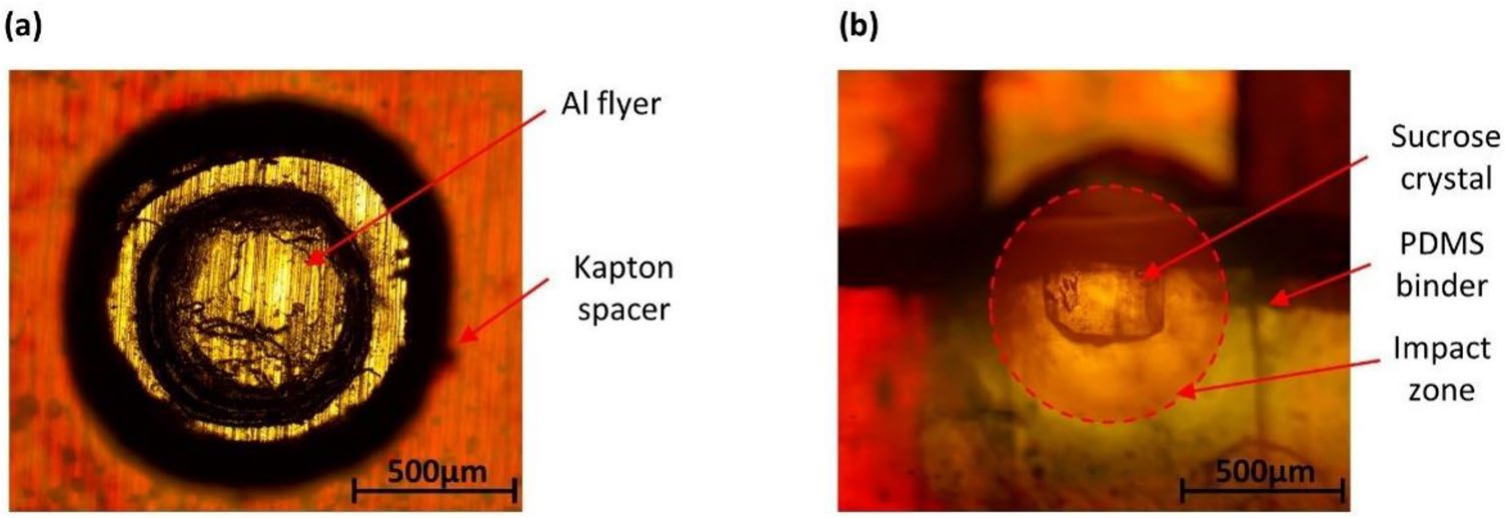
Preparation of launchpad for shock compression experiments where (**a**) Aluminum flyer enclosed by 300 µm Kapton spacer is used for impacts and (**b**) assembled with the target as sucrose crystal overlayed on the impact zone.

In this work, the high-velocity impact is produced using a laser-based projectile launch setup. The laser-based projectile launch system used in this work is a modified version of the system used in previous work^2,33,46^. The details of modifications are included in the supplementary datasheet. A Nd: YAG laser from Continuum Lasers was used with a pulse width of 7 ns centered at 1064 nm wavelength and maximum pulse energy of 650 mJ. The launch assembly for the aluminum flyer was prepared with a thin aluminum foil (AL1100) from Alufoil Products Co., Inc. glued to a 3 mm thick borosilicate glass slide from McMaster-Carr Co. The two-part epoxy from Loctite (Ablestik 24) is used for adhesive bonding of 25 µm and 50 µm aluminum foil to the glass slide. The aluminum foils were machined in form of a circular disk of 600 µm diameter using laser machining to improve the consistency of launch. The launchpad is completed by attaching a 300 µm Kapton separator as shown in Fig. 7a. The Kapton spacer was also made by cutting a circular disk of 900 µm diameter using laser machining. The PBS samples were assembled in front of the aluminum disk as shown in Fig. 7b and the whole assembly was integrated inside a vacuum chamber. A vacuum of around -0.85 Bar was applied to reduce air drag while performing impact experiments.

### In-situ time-resolved Raman spectroscopy

The Raman signal is excited by a Nd: YAG pulsed laser source at 532 nm and pulse duration of ~ 20 ns. For shift measurements, a 75 μm vertical entrance slit on the 0.5 m focal length spectrograph from Princeton Instruments (Acton SP2500) is used. The spectrograph is integrated with the Hamamatsu streak camera (C4334-01) where the Raman spectrum is acquired over a 50 ns window. The horizontal photocathode slit on the streak is 70 μm and a spectrum of 15.76 nm is captured on the CCD with a resolution of 0.15 nm. A fixed delay is added between 532 laser and 1064 nm laser based on the estimated shock arrival time in the sucrose crystal. The laser pulse from the flyer launch laser and Raman probe laser are triggered using the single-shot feature on the delay generator. The uncertainty between the time of collecting Raman signal and the arrival of shock wave at sugar crystal is discussed in the supplementary datasheet.

### Measurement of pressure, temperature, and shock viscosity

The shock compression of solids can lead to temperature rise and chemical or structural changes. Therefore, separation of temperature effects on Raman shift is important to obtain the correct analysis of shock pressure. Hare et al.^3^ used a combined effect of temperature and pressure to Raman shift, providing an estimate of shock pressure and temperature in thin PMMA films under ablation heating. The work of Gan et al.^47–49^ suggests that the effects of temperature and stress on Raman shift can be assumed independent of each other under isothermal conditions. The work of Zhang et al.^50–54^ separated the effects of stress and temperature on Raman shift by calibration of peak width with temperature. Through measurement of peak width, the estimated temperature was used to correct for temperature-induced peak shift. However, the use of peak width for temperature estimation requires a constant exposure time and laser energy under calibration and experiments. In a dynamic experiment such as shock compression, the variation of laser energy over laser pulse width and spectra collected over a large number of experiments can lead to uncertainty in peak width measurements. Therefore, measurement of peak location was preferred in this work. A simple approach is used to express the change in Raman shift as a function of pressure and temperature as defined in Eq. (2). These equations are used with an assumption that mixed cross-terms such as $\frac{{\partial^{2} \omega }}{\partial P\partial T} = 0$

2 $$\Delta \omega \left( {P,\Delta T} \right) = \Delta \omega \left( P \right) + \Delta \omega \left( {\Delta T} \right)$$

3 $$\Delta \omega_{{CH_{2} }} \left( {P,\Delta T} \right) = \left( {6.35} \right) \times P \left[ {{\text{GPa}}} \right] - \left( {1.2 \times 10^{ - 2} } \right) \times \Delta T \left[ {^\circ {\text{C}}} \right]$$

4 $$\Delta \omega_{CH} \left( {P,\Delta T} \right) = \left( {9.85} \right) \times P \left[ {{\text{GPa}}} \right] - \left( {2.2 \times 10^{ - 2} } \right) \times \Delta T \left[ {^\circ {\text{C}}} \right]$$

**Figure 8:**
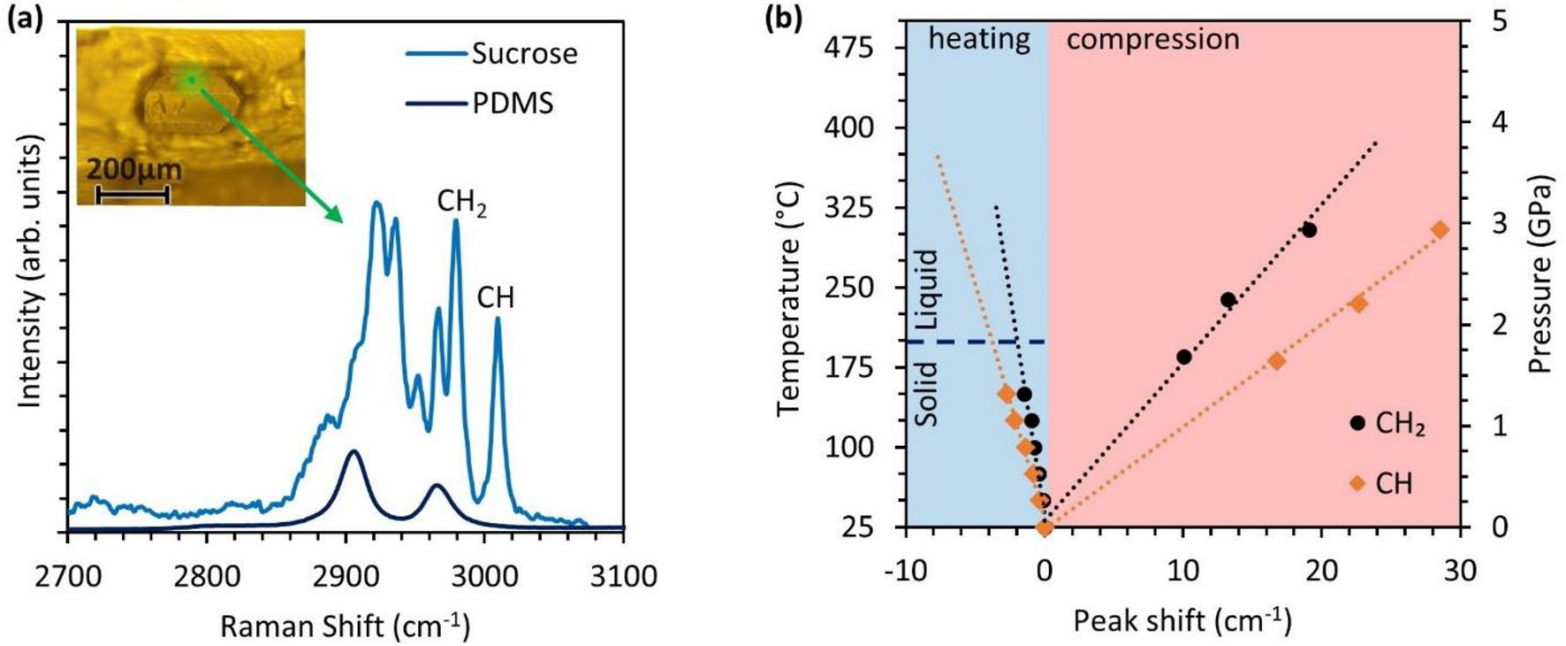
Calibration of change in Raman shift with temperature and pressure where (**a**) shows the Raman Spectra of PDMS polymer and sucrose crystal and (**b**) shows the behavior of CH_2_ and CH functional groups in sucrose with pressure and temperature.

The solution for pressure and temperature can be obtained by solving a system of equations using the behavior of multiple functional groups. In this work, the Raman probe laser is focused on a 22 μm region ^2^ on the sucrose crystal and vicinity of the interface between sucrose crystal and PDMS as shown in Fig. 8a and further explained in Supplementary Fig. s7. The behavior of functional groups in sucrose between 2900 and 3100 cm^−1^ is captured under shock compression. The CH_2_ group at 2982 cm^−1^ and CH group at 3011 cm^−1^ ^42,55^ are analyzed due to ease in locating peaks during data processing. The behavior of these groups as a function of pressure is obtained from the work of Ciezak-Jenkins et al.^42^. The behavior with temperature rise is calibrated on a hot stage designed to control the temperature within ± 1 °C. Sucrose crystals are embedded inside high-temperature cement to provide uniform heating and Raman spectra were collected for 15 min after the temperature stabilization for phase stabilization. Further details on temperature calibrations are included in the supplementary datasheet. The calibrations are achieved through a linear fit as shown in Fig. 8b and provided in Eqs. (3) and (4). The time-resolved Raman spectra during shock loading provide an estimate of shock pressure rise time. The obtained values of rise time and pressure are used to estimate shock viscosity^38^,

5 $$\eta = \frac{1}{4}S\sigma_{h} \tau$$

where S is the slope of shock velocity to particle velocity relationship ^34,35^, $\sigma_{h}$ is the Hugoniot state stress and $\tau$ is shock rise time.

## Supplementary Information

Supplementary Information.

## Data Availability

The datasets generated during and/or analyzed during the current study are available from the corresponding author on reasonable request.
