# Supplementary material for: Thermo-mechanical behavior measurement of polymer-bonded sugar under shock compression using in-situ time-resolved Raman spectroscopy
Source: Sci Rep. 2022 Feb 3;12:1876. doi: 10.1038/s41598-022-05834-3 (PMC8814196; doi:10.1038/s41598-022-05834-3)
Supplement: Supplementary file 1 — Supplementary Information. [file 41598_2022_5834_MOESM1_ESM.pdf]

Thermo-mechanical behavior measurement of polymer-bonded sugar under shock compression using *in-situ* time-resolved Raman spectroscopy

Abhijeet Dhiman[1], Nolan S. Lewis[1], Ayotomi Olokun[1], Dana D. Dlott[2], Vikas Tomar[1]

[1] School of Aeronautics and Astronautics, Purdue University, West Lafayette, Indiana 47907, USA

[2] School of Chemical Sciences and Fredrick Seitz Materials Research Laboratory, University of Illinois at Urbana-Champaign, Urbana, Illinois 61801, USA

Experiment Method

Sample preparation

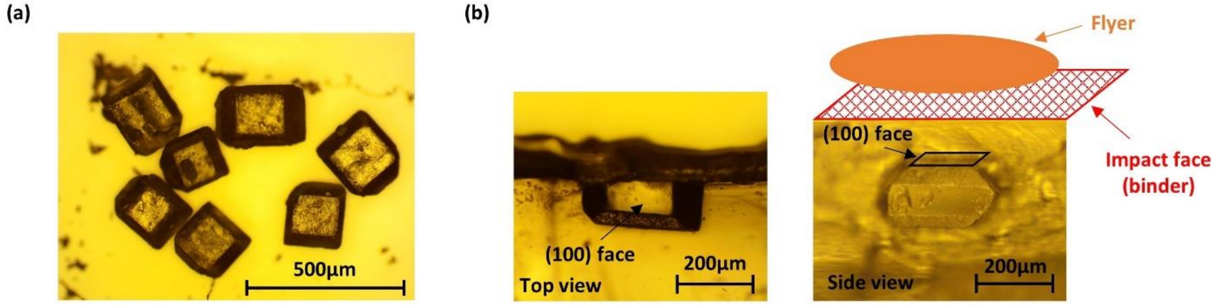

Supplementary Figure S1: (a) Sucrose crystals used for sample preparation (b) Top (left) and side (right) view of the sucrose crystal embedded inside the PDMS binder.

In this work, the samples were created using single-crystal sucrose particles, Supplementary Figure S1(a), with an average crystal size of 250 μm. The sucrose crystals were embedded inside PDMS binder with a controlled orientation such that (100) orientation, also most prominent during growth [1], is oriented perpendicular to the impact direction and embedded with an average depth of 106.6 μm and standard deviation of 10.5 μm inside PDMS binder (Sylgard 184, Dow Chemical Company), Supplementary Figure S1(b). In order to reduce the contribution of functional groups in PDMS to the Raman shift measurements, samples were prepared without the PDMS binder on the side face enabling the collection of Raman spectra from sugar crystal.

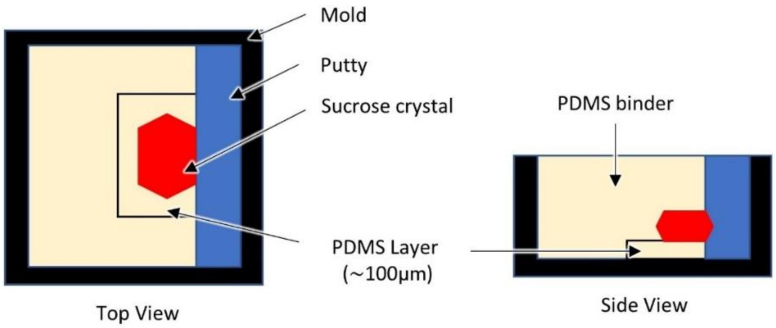

Supplementary Figure S2:Schematic view of sample preparation method for the embedding of sucrose crystal inside PDMS binder.

To achieve this, we used a two-step process for embedding crystals in the binder. First, a 100 µm thick layer of PDMS was formed on a glass surface using spin coating which provides a mean to control the depth of crystal inside the binder. The deposited layer was cured at 80 ºC for 4 hours. Later, this layer was used as a base to place sucrose crystals inside the mold as shown in Supplementary Figure S2. The mold was prepared with adhesive putty on one side. The sucrose crystal was placed carefully with face (100) on the thin PDMS layer and touching the adhesive putty on the other side. The adhesive putty act as a barrier to the PDMS binder and provides a surface free of the binder. Finally, the PDMS binder was poured into the mold to complete the embedment followed by curing at 80 ºC for 4 hours. Once fully cured, the adhesive putty is removed and samples were imaged under a microscope to verify the quality of sample preparation. Supplementary Figure S3 shows a few samples selected for experiments. Overall, an average depth of 106.6 µm and a standard deviation of 10.5 µm inside the PDMS binder were achieved in these experiments (Supplementary Figure S4).

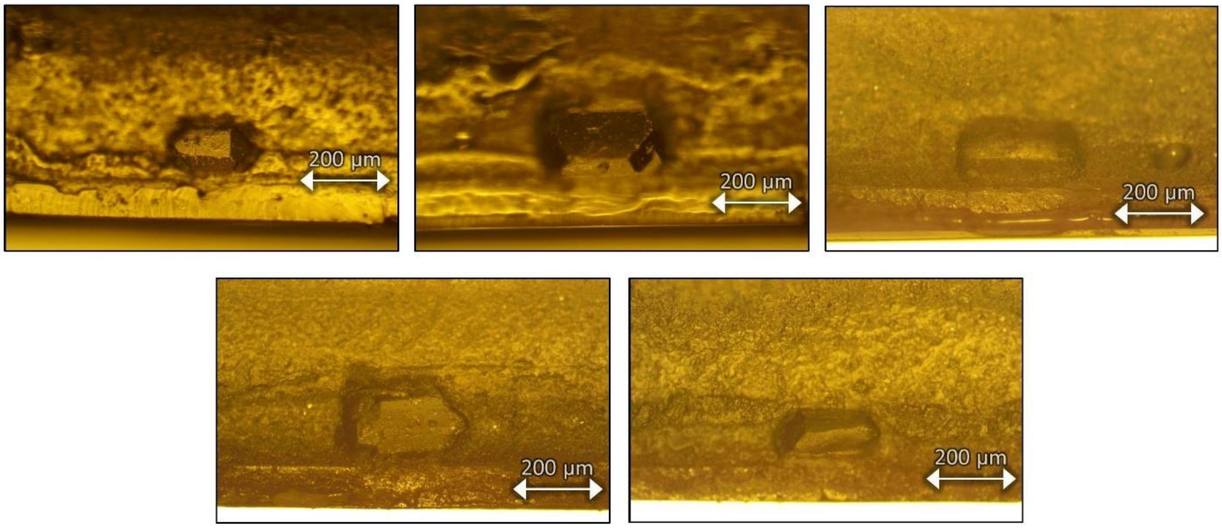

Supplementary Figure S3:Samples with sucrose crystals embedded 100 µm inside the PDMS binder.

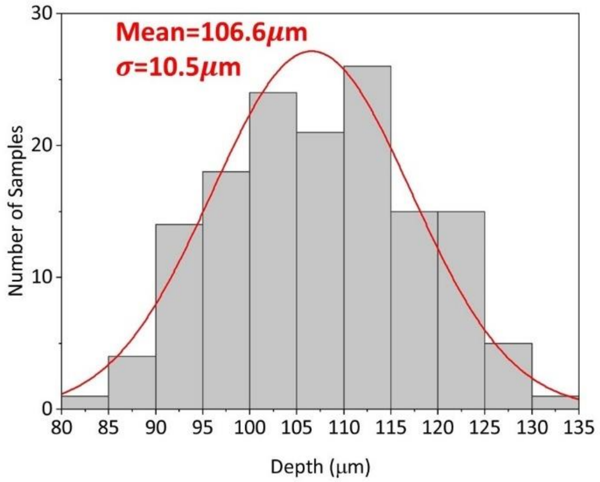

Supplementary Figure S4: Distribution of the depth of sucrose crystals embedded inside the PDMS binder.

Laser-based projectile launch setup

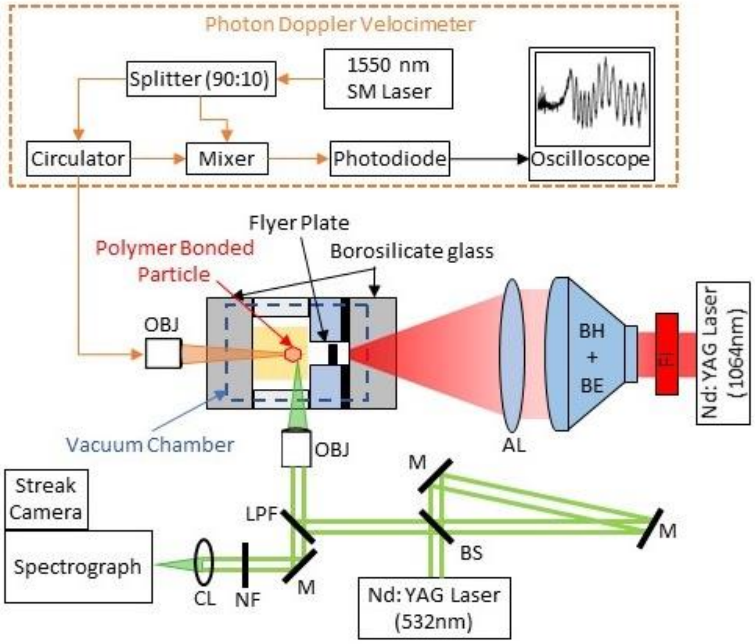

Supplementary Figure S5: Schematic of the laser-based projectile launch system with in-situ Raman spectroscopy and photon Doppler velocimetry (PDV). LPF = long pass filter, NF = notch filter, M = mirror, BS = beam splitter, BE = beam expander, BH = beam homogenizer, OBJ = objective lens, CL = camera lens, AL = aspheric lens, FI = farady isolator.

The laser-based projectile launch system used in this work is a modified version of the system used in our previous work [2]. The principle behind such a mechanism of accelerating projectile to high speeds involves using a pulse laser beam to drive thin metal foils. A Nd: YAG laser from Continuum Lasers was used with a pulse width of 7 ns centered at 1064 nm wavelength and maximum pulse energy of 650 mJ. The flash lamp of the laser runs continuously at 10 Hz and a single pulse was triggered using a delay

generator (Stanford Research Systems, Model - DG645) in synchronization to Nd: YAG – 532 nm laser, streak scope, and PDV system. The beam profile of the laser was modified from Gaussian to flat-top using diffractive optics from HOLO/OR Ltd. The PDV system shown in Supplementary Figure S5 earlier is the same as our previous work [2].

In order to obtain a planar impact profile from thin metal foils, a spatially homogenized laser spot was created at the launch assembly using a diffuser element from HOLO/OR Ltd (RD-204-I-Y-A). The beam was expanded from 8 mm to 45 mm using a system of telescopic lenses and a spatially homogenized spot of diameter 800 µm was achieved after focusing through an aspheric lens of 150 mm focal length (AL75150-C, Thorlabs Inc.). The beam profile before and after using diffuser optics is shown in Supplementary Figure S6(a) and (b) respectively.

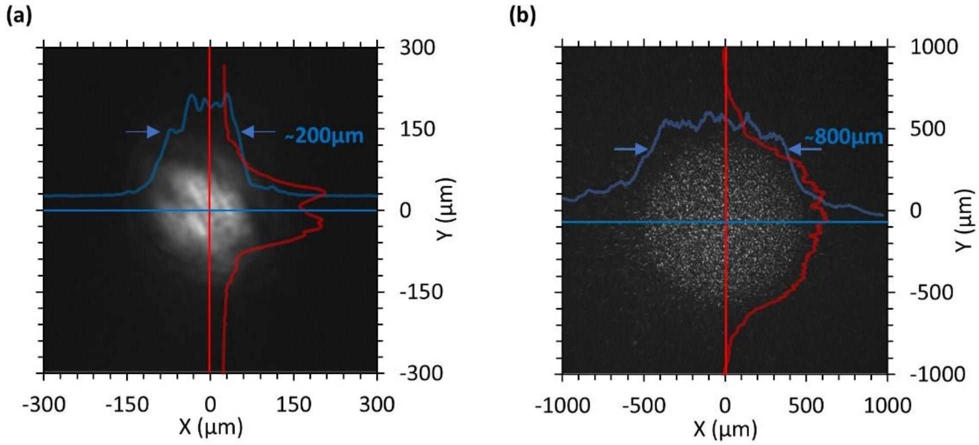

Supplementary Figure S6: (a) Spatial profile of the Nd:YAG (1064nm) laser output beam (b) Spatial profile of the Nd:YAG (1064nm) laser with diffuser optics

In-situ time-resolved Raman spectroscopy

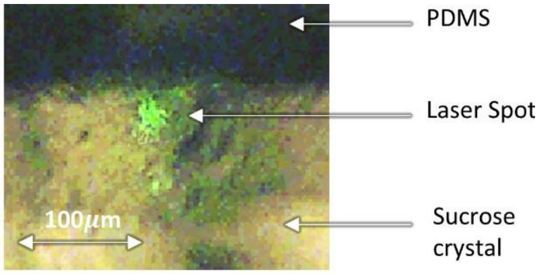

Supplementary Figure S7: 532 nm laser spot on the sample. The spot is placed on the sucrose crystal in the proximity of the sucrose-PDMS interface.

The Raman signal is excited by a Nd: YAG pulsed laser source at 532 nm and pulse duration of ∼10 ns. The laser is focused on sucrose crystal in the proximity of interface between sucrose crystal and PDMS binder as shown in Supplementary Figure S7. The laser spot diameter of $\sim 22\ \mu m$ can be observed on the crystal and depth of field of $12.4\ \mu m$ can be estimated using equation (1) for subsurface measurement[3].

$$d.o.f = \frac{n\lambda}{NA^2}, \qquad (1)$$

where n is the refractive index of the medium between the sample and the objective (1.46 for quartz window), λ is laser wavelength (532 nm) and NA is the numerical aperture of the objective (0.25 for LMH-10X-532 - Thorlabs). The pulse width of this laser was stretched using an external 10 m cavity and 60:40 beam splitter as shown in Supplementary Figure S5. Supplementary Figure S8(a) shows the Raman shift corresponding to the C-H function groups (2900 cm^{-1}-3100 cm^{-1}) in sucrose measured using stretched 532 nm pulse laser. The Raman shift corresponding to CH_2 (2982 cm^{-1}) and CH (3011 cm^{-1}) functional groups [4,5] are used for pressure and temperature measurement. Supplementary Figure S8(b) shows the temporal intensity profiles of the two functional groups where a pulse width of 19.6 ns was observed after stretching through the external cavity.

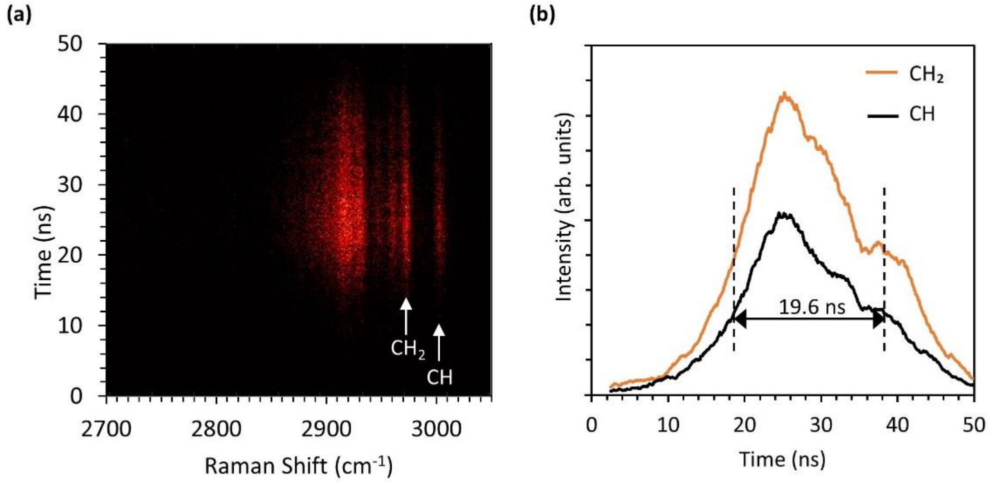

Supplementary Figure S8: (a) Time-resolved Raman spectrum from sucrose crystal (c) Temporal profile of CH and CH_2 functional groups in sucrose using 20 ns laser pulse

Calibration of Raman shift with temperature

The calibration of the change in Raman shift for sucrose with temperature was performed using the Raman microscope from Horiba Scientific combined with a hot stage. As shown in Supplementary Figure S9(a), the sucrose crystals were embedded inside high-temperature cement (Omega Engineering, Inc.) to ensure uniform heating of the crystals. The Raman spectra were collected with 532 nm laser and 5 mW of laser power accumulating over 10s. A slit size of 25 µm was used to obtain Raman spectra. As shown in

Supplementary Figure S9(b), the Raman spectra from sucrose were observed till 150 °C where spectra were collected after 15 min of reaching a stable temperature. The behavior of CH_2 and CH functional groups is shown in Supplementary Figure S9(c).

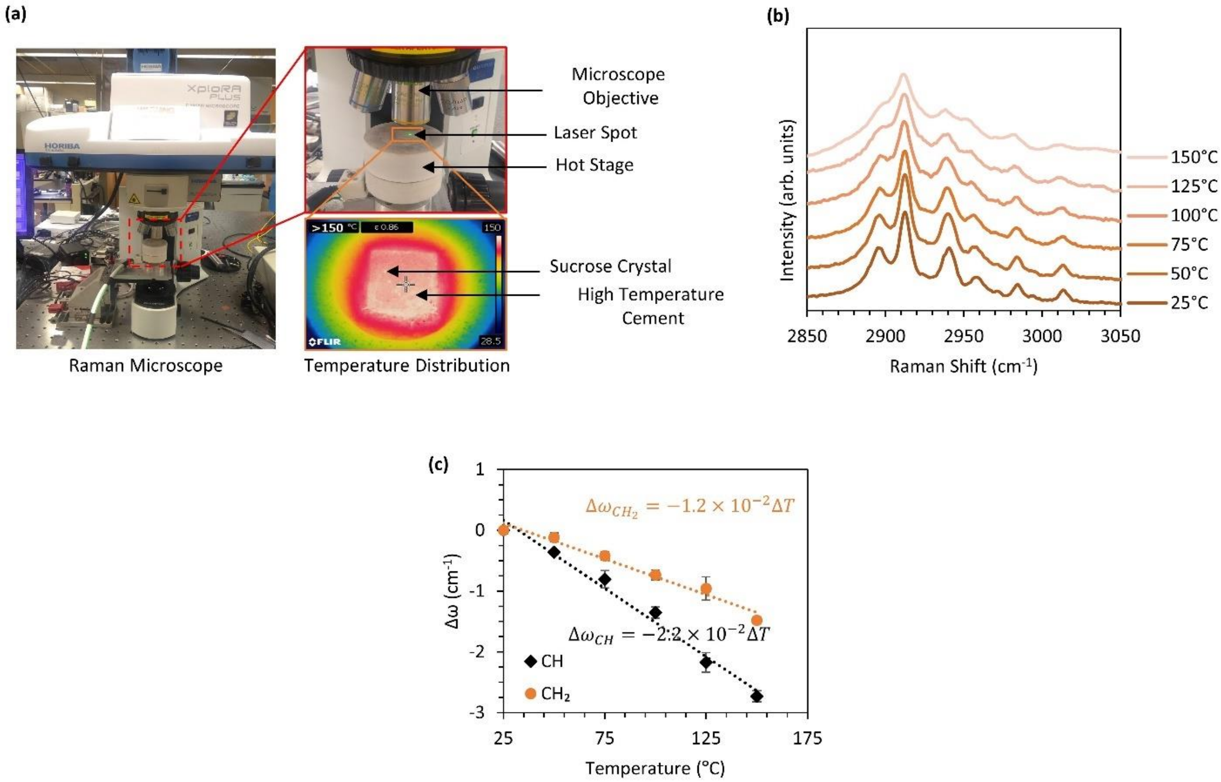

Supplementary Figure S9:(a) Raman microscope system combined with a hot stage for temperature calibration of Raman shifts (b) Measured Raman spectra from sucrose as a function of temperature (c) Measured change in Raman shift for CH_2 and CH functional groups in sucrose as a function of temperature.

Results

Flyer impact characterization

Supplementary Figure S10(a) shows images of flyers recovered after the impact on the glass target at impact speeds of 1.2 km/s and 0.62 km/s. These flyers of thickness 25 μm and 50 μm were launched at 650 mJ. Supplementary Figure S10(b) shows a velocity history obtained through PDV for a 25 μm flyer during impact on the glass target. The oscillations observed during the acceleration of the flyer are attributed to the reverberating shock in the flyer during launch [6]. Such reverberation can be reduced by expanding the pulse width of the launch laser. However, in this work, a 300 μm spacer was found sufficient for a flyer to reach stable impact velocity. The velocity history profile also shows particle velocity in glass (U_p) where shock duration of ~ 3 ns and ~ 8 ns were observed for impacts with 25 μm and 50 μm flyers. The flyer

velocity (U_{impact}) for *in-situ* Raman spectroscopy experiments performed using 25 μm and 50 μm flyer were observed to be $1.2 \pm 0.12\ km/s$ and $0.62 \pm 0.06 km/s$ respectively (Supplementary Figure S11).

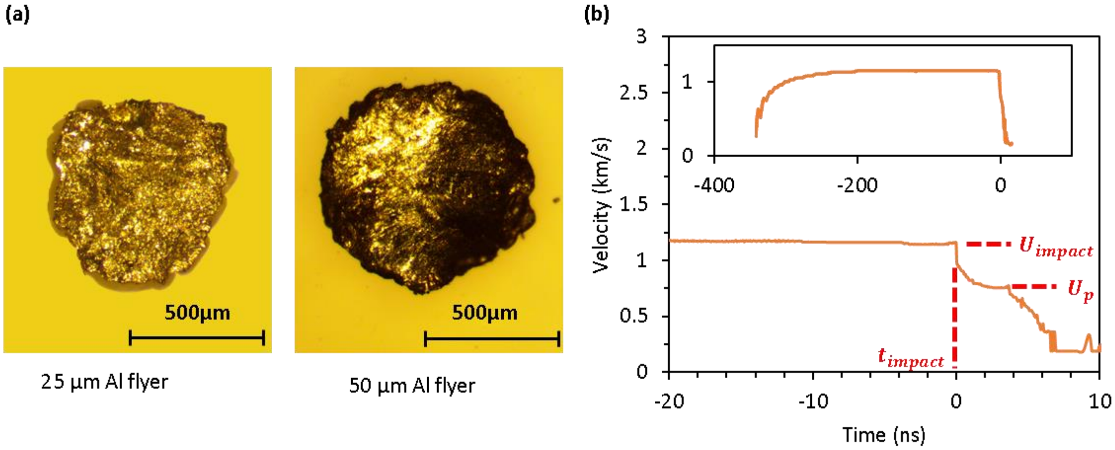

Supplementary Figure S10: (a) Images of 25 μm (left) and 50 μm (right) aluminum flyers recovered after impact on a glass target at ~1.2 km/s and ~0.65 km/s respectively (b) Velocity profile for 25 μm flyer during impact on the glass target obtained using PDV.

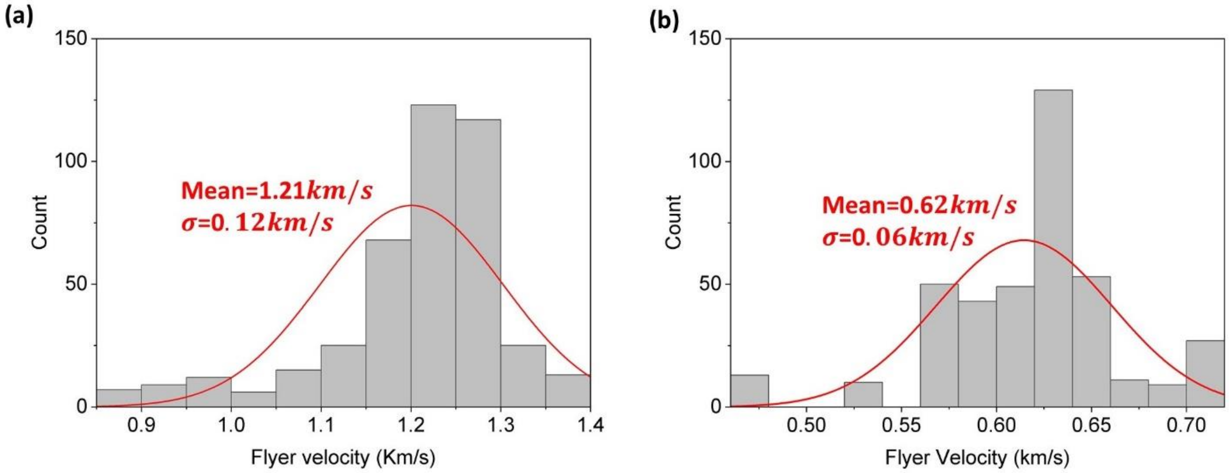

Supplementary Figure S11: Distribution of flyer velocity for experiments leading to shock compression of (a) 4.26 GPa and (b) 2.22 GPa

Analysis of Time-resolved Raman spectra under shock compression

The streak data was binned over a 5 ns window to resolve Raman shifts and obtain peak location using multiple Lorentz peak fitting in Origin Pro (OriginLab Corporation). The results of peak fitting for $–CH_2$ and –CH stretching vibration region (2800-3100 cm^{-1}) [7,8] are shown in Supplementary Figure S12 for two impact velocities leading to shock pressure of 4.26 GPa and 2.22 GPa. Several changes in Raman spectra can be observed during the rise of shock pressure. Most noticeable are the broadening and splitting of CH_2 and CH functional groups to a higher Raman shift around 45 ns after impact. The broadening of Raman shifts is associated with the temperature rise during shock compression and the splitting of the peaks to a

higher shift is associated with the increased pressure. The movement of CH_2 and CH functional groups is shown using blue and purple colored peaks for easier identification.

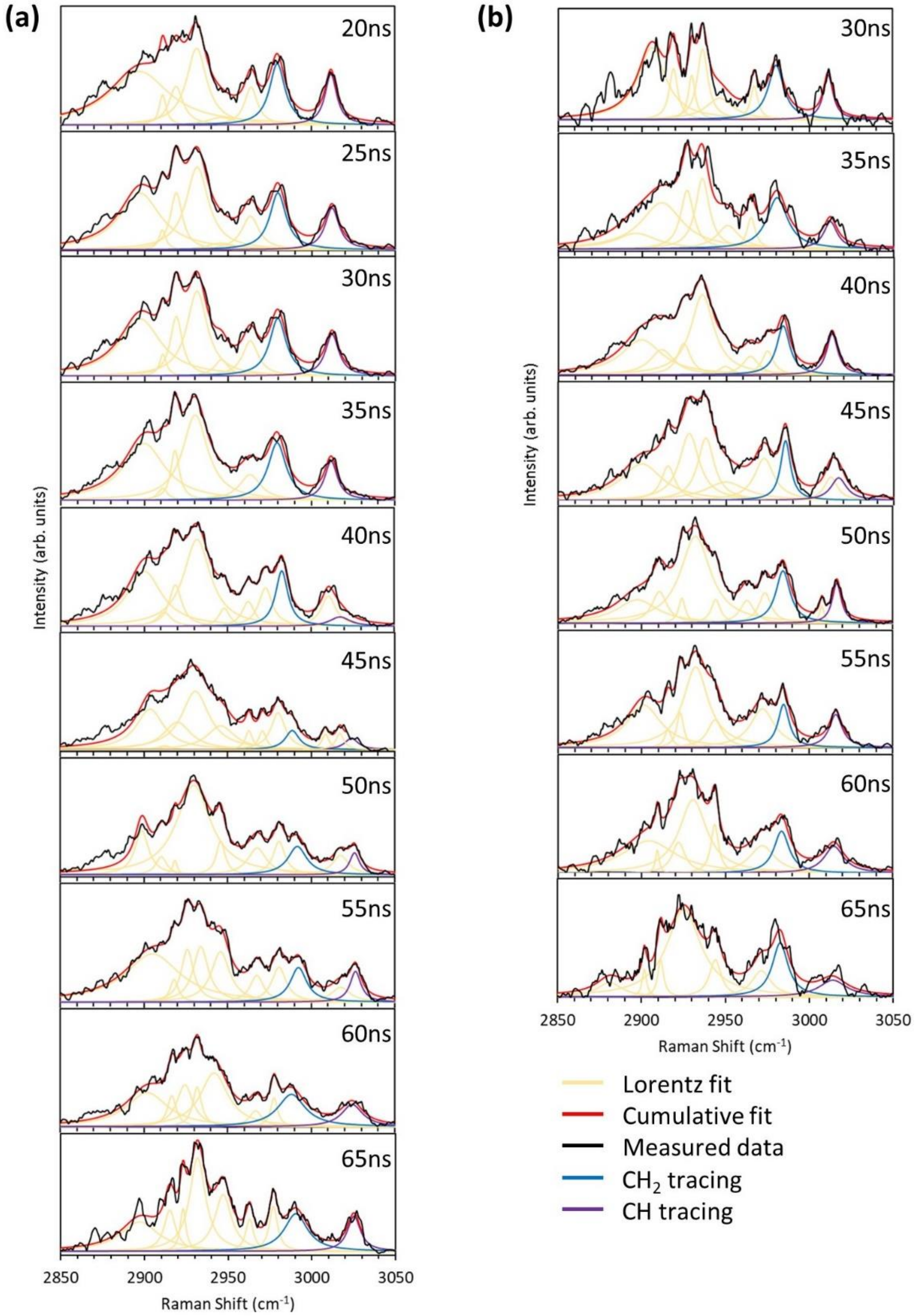

Supplementary Figure S12: Multi-peak Lorentz fitting of Raman spectra of sucrose during shock compression of (a) 4.26 GPa and (b) 2.22 GPa.

It is important to note that these Raman spectra are a combination of ∼400 impacts performed over moving laser window due to uncertainty related to the time of impact on the sample. The uncertainty in the time of impact has contributions from the uncertainty in flyer velocity and the distance between the flyer to sample surface. The overall variation in the time of impact for two experiment settings is shown in Supplementary Figure S13. The time for probing Raman spectrum from the sample is fixed at a delay of 40/50 ns with respect to impact time of 0 ns (synchronized with flyer launch laser) for two impact velocities. The time of delay was calculated based on the time of travel for the shock front through the PDMS layer (thickness 106μm). Therefore, Raman spectra collected for each experiment were corrected for the estimated arrival time of shock wave in the probed region based on the time of impact. The uncertainty related to the arrival time of the shock wave in the probed region is given in the next section.

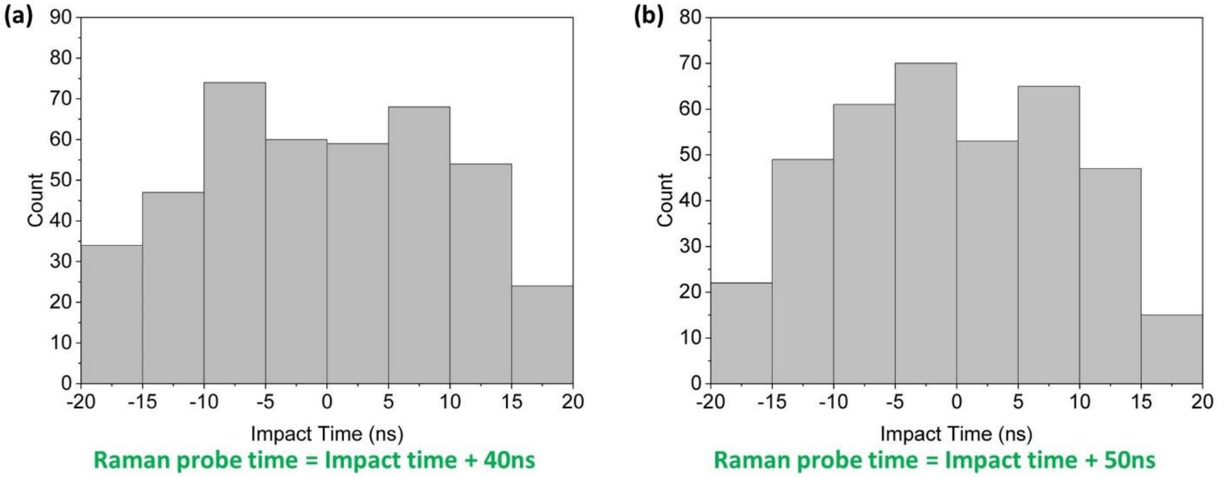

Supplementary Figure S13: Time of impact on the PBS sample observed for experiments leading to shock compression of (a) 4.26 GPa and (b) 2.22 GPa.

Uncertainty analysis for the design of the experiment

The major components for the uncertainty in the arrival of shock wave at the interface of PDMS-sucrose are the thickness of the binder layer (Supplementary Figure S4), Flyer velocity (Supplementary Figure S11), and the time of impact (Supplementary Figure S13). The uncertainty related to the time of impact was corrected on post-processing of data using velocity profile from PDV. The time resolution of 1 ns was used for STFT during the processing of the PDV signal. Therefore, the arrival time of shock wave at the PDMS-sucrose interface can be expressed as,

$$t_{shock} = t_{impact} + \frac{h_{binder}}{U_s} \tag{2}$$

where h_{binder} is the thickness of the PDMS layer, U_s is the shock speed in HTPB and t_{impact} is the time of impact on the sample. The total uncertainty for the arrival time of shock wave at the interface can be expressed as,

$$\Delta t_{shock} = \sqrt{\Delta t_{impact}^2 + \left[\frac{h_{binder}}{U_s} \times \sqrt{\left(\frac{\Delta h_{binder}}{h_{binder}}\right)^2 + \left(\frac{\Delta U_s}{U_s}\right)^2}\right]^2} \quad (3)$$

where, $\Delta t_{impact} = 1$ ns (uncertainty from PDV analysis), $\Delta h_{binder} = 10.5$ μm. The uncertainty in U_s was calculated in Engineering Equation Solver based on impedance matching between aluminum 1100 flyer [9] and PDMS binder [9]. The calculated shock speed for the flyer velocity of 1.21±0.12 km/s and 0.62±0.06 km/s are 3.28±0.15 km/s and 2.5±0.08 km/s respectively. The total uncertainty of 3.7 ns and 4.5 ns is estimated for flyer velocity of 1.21 km/s and 0.62 km/s respectively. Therefore, a maximum resolution of ~5 ns for time-resolved Raman spectroscopy can be obtained in this work.

References:

1 Sgualdino, G., Aquilano, D., Tamburini, E., Vaccari, G. & Mantovani, G. On the relations between morphological and structural modifications in sucrose crystals grown in the presence of tailor-made additives: effects of mono-and oligosaccharides. *Materials chemistry and physics* **66**, 316-322 (2000).

2 Dhiman, A., Olokun, A. & Tomar, V. Microscale Analysis of Stress Wave Propagation through Plastic Bonded Explosives under Micro-Sphere Shock Impact. *Journal of Dynamic Behavior of Materials* (2021).

3 Shillaber, C. P. Photomicrography in theory and practice. (1944).

4 Brizuela, A. B. *et al.* A complete characterization of the vibrational spectra of sucrose. *Carbohydrate research* **361**, 212-218 (2012).

5 Ciezak-Jenkins, J. A. & Jenkins, T. A. Mechanochemical induced structural changes in sucrose using the rotational diamond anvil cell. *Journal of Applied Physics* **123**, 085901 (2018).

6 Curtis, A. D., Banishev, A. A., Shaw, W. L. & Dlott, D. D. Laser-driven flyer plates for shock compression science: Launch and target impact probed by photon Doppler velocimetry. *Review of scientific instruments* **85**, 043908 (2014).

7 Szostak, M. M., Piela, K., Hołderna-Natkaniec, K., Natkaniec, I. & Bidzińska, E. Optical nonlinearity and electric conductivity origin study on sucrose crystal by using IR, Raman, INS, NMR, and EPR spectroscopies. *Carbohydrate research* **395**, 29-37 (2014).

8 Kishore, R. & Padmanabhan, V. in *Proceedings of the Indian Academy of Sciences-Section A.* 360 (Springer).

9 Marsh, S. P. *LASL shock Hugoniot data*. Vol. 5 (Univ of California Press, 1980).
